# Reduction of Tendon Adhesions following Administration of Adaprev, a Hypertonic Solution of Mannose-6-Phosphate: Mechanism of Action Studies

**DOI:** 10.1371/journal.pone.0112672

**Published:** 2014-11-10

**Authors:** Jason K. F. Wong, Anthony D. Metcalfe, Richard Wong, Jim Bush, Chris Platt, Arnaud Garcon, Nick Goldspink, Duncan A. McGrouther, Mark W. J. Ferguson

**Affiliations:** 1 Plastic Surgery Research, Faculty of Medicine and Human Sciences, University of Manchester, Manchester, United Kingdom; 2 Renovo Ltd, Core Technology Facility, Manchester, United Kingdom; National Institutes of Health, United States of America

## Abstract

Repaired tendons may be complicated by progressive fibrosis, causing adhesion formation or tendon softening leading to tendon rupture and subsequent reduced range of motion. There are few therapies available which improve the gliding of damaged tendons in the hand. We investigate the role of Mannose 6-phosphate (M6P) in a 600 mM hypertonic solution (Adaprev) on tendon adhesion formation *in viv*o using a mouse model of severed tendon in conjunction with analysis of collagen synthesis, cellular proliferation and receptors involved in TGF beta signalling. Cytotoxicity was assessed by measuring tissue residency, mechanical strength and cell viability of tendons after treatment with Adaprev. To elicit potential modes of action, *in vitro* and *ex vivo* studies were performed investigating phosphorylation of p38, cell migration and proliferation. Adaprev treatment significantly (p<0.05) reduced the development of adhesions and improved collagen organisation without reducing overall collagen synthesis following tendon injury *in vivo*. The bioavailability of Adaprev saw a 40% reduction at the site of administration over 45 minutes and tendon fibroblasts tolerated up to 120 minutes of exposure without significant loss of cell viability or tensile strength. These favourable effects were independent of CI-MPR and TGF-β signalling and possibly highlight a novel mechanism of action related to cellular stress demonstrated by phosphorylation of p38. The effect of treatment reduced tendon fibroblast migration and transiently halted tendon fibroblast proliferation *in vitro* and *ex vivo*. Our studies demonstrate that the primary mode of action for Adaprev is potentially *via* a physical, non-chemical, hyperosmotic effect.

## Introduction

Repaired tendons may be complicated by the paradoxical problems of fibrosis, causing adhesion formation, and tendon softening, causing tendon rupture and/or reduced range of motion. Numerous therapies have been investigated with the aim of improving the gliding function of damaged tendons in the fingers [1]. In England between 2012 and 2013, 17555 primary tendon repairs were performed together with 3537 tendon freeing procedures (tenolysis) as a result of adhesions [2]. The average length of treatment in splint is 6 weeks and estimated time to full functional recovery around 12 weeks [3]. Around 28% [4] to 57% [5] of patients have a fair to poor functional recovery after flexor tendon surgery and failed repairs account for 3.9% [6] to 30% of patients [7]. Although there has been a recent trend to advocate cell based [8] and growth factor directed therapies [9] in tendon injuries few strategies have been adopted clinically.

Wound healing and the process of scar formation is a mammalian response to injury that applies to many tissues [10] including flexor tendon healing. Adhesion formation between the sheath and tendon arises from a combination of cellular proliferation and collagen deposition within the surrounding injured tissue, restricting gliding function that peaks at around three to four week and matures by eight weeks [*11*]. Transforming growth factor beta 1 (TGF-β1) has been implicated in adhesion formation [12], and manipulating TGF-β via neutralising antibodies post-surgery reduces the number and size of adhesions [13]. Mannose-6-Phosphate (M6P) has been demonstrated to reduce active TGF-β1 expression on cultured tendon fibroblasts and improved range of movement in a rabbit flexor tendon injury model [14]. Studies of M6P in relation to skin scarring also demonstrate improvement in scar cosmesis and accelerated return of normal dermal architecture [15]. However the mechanism by which M6P reduces adhesion formation is still unclear and it is questionable whether its mode of action is via the inhibition of the TGF-β1 pathway. Indeed, TGF-β1 and its receptors are only expressed at significant levels 7 to 28 days after injury [12], [16] but the administration time frame of M6P in studies are inconsistently earlier [14]. It has also been established that latent TGF-β is activated by a range of CI-M6PR independent mechanisms [17] and that mannose phosphorylation has little role in inhibiting the activation of TGF-β1 [18], which indicates there may be other mechanisms for M6P to elicit its antiscarring effect [19], and anti-adhesion effect [14], [20]. Thus, we set out in this study to elicit whether M6P was effective at reducing tendon adhesions and if so by which biological effects and by which potential mechanisms.

## Materials and Methods

### Preparation of Mannose 6-Phosphate and Glucose 6-Phosphate

Mannose 6-Phosphate (M6P; Sigma-Aldrich, UK) was originally prepared for study using 14 mg/ml, 56 mg/ml and 169 mg/ml to produce 50 mM, 200 mM, and 600 mM solutions respectively.

Mannose 6-Phosphate (M6P; Sigma-Aldrich, UK), or Glucose 6-Phosphate (G6P; Sigma-Aldrich, UK) was weighed to make up a 600 mM (169.26 mg/mL) solution, which was then placed into a volumetric flask and Phosphate buffered saline (PBS, Life Technologies, USA.) added (approximately 2 mL less than the total desired volume was prepared to allow for neutralisation). The solution was inverted several times to aid dissolution. A 100 µL pipette (Gilson, UK) was used to slowly add 10M Sodium Hydroxide (NaOH; Sigma-Aldrich, UK) drop wise to the solution, swirling after each addition, until the solution was neutralised. The solution was allowed to stand at room temperature for 30 min to allow any remaining M6P or G6P to dissolve. After 30 minutes, the pH of the solution was determined and adjusted to pH 7.0 (±0.5) using 10M NaOH. From this stock solution dilutions were made to prepare 50 mM, 200 mM and 600 mM solutions using PBS.

In subsequent studies osmolality was checked at 150 mM, 300 mM and 600 mM using a 3320 Micro-osmometer (Advanced Instruments Inc, USA.) and preparations specifically of 50 mM, 200 mM and 600 mM (Adaprev) were used for study.

### Solution distribution study

Ten mouse digits had 2 µL of 1∶50 Vybrant DiI solution (Molecular Probes, USA) administered into the flexor tendon sheath under 20x magnification. Five mice were harvested immediately after wound closure and five were harvested one day after administration of DiI. Following fixation, decalcification, wax processing and serial sectioning, images were captured using a SPOT camera (Diagnostic instruments Inc, USA) mounted on a Leica DMRB microscope (Leica Microsystems, Germany) using a 5x objective. Images were uploaded into a 3D reconstruction program (Reconstruct) [21] and a 3D representation of solute distribution was produced.

### Therapeutic study

The effect of treatment was reviewed at three weeks following injury, the point of greatest fibroblast activity and adhesion deposition, and also reviewed at eight weeks coinciding with the end of the synthetic phase [11].

Reconstituted M6P at doses 50 mM (low), 200 mM (moderate) or 600 mM (high; Adaprev) were used for different treatment groups. Recombinant human TGF-β1 (R&D systems, USA.) was used at a concentration of 10 nM. This was reconstituted in sterile 4 mM Hydrochloric acid (HCl; Sigma-Aldrich, UK) and 0.1% human serum albumin solution (Sigma-Aldrich, UK) and selected for its pro-fibrotic effects as a positive control. This dose was selected from dosage studies performed on skin wounds in rats [22]. Normal 0.9% saline was used on the contralateral wounded limb as a control. The allocation of treatment to each mouse digit was performed in a single blinded randomised fashion to minimise study bias and the study designed with n = 5 per treatment at each time point.

### Mice Operative model

All work was approved by the Local Ethical Review Committee at the University of Manchester, and complied with British Home Office regulations on care and use of laboratory animals (Project licence Number PPL 40/2734). Our previously described adhesion model [11] was used to assess the effects of Adaprev therapy.

The mouse *in vivo* study used the hindpaw deep digital flexor of male C57/BL6 mice aged between 10 and 12 weeks (25–30 g) (Charles River, UK). Surgery was performed under a standard mouse general anesthetic protocol (Induction using 4% isoflurane (Abbott, UK) and 4 l/min oxygen driver, maintenance 2% isoflurane with 2 l/min oxygen driver and 1.5 l/min nitrous oxide [BOC healthcare, UK).

Under tourniquet control a partial laceration wound was performed that involved approximately 50% of hindpaw flexor tendon fibres. Depending on the study allocation, wounds were treated with either 0.9% saline, PBS, TGF-β1 10 nM or M6P (n = 5 per treatment group) at 50 mM, 200 mM or Adaprev, infiltrated into the sheath. A proximal tenotomy was performed to avoid the tendon reuniting and minimise tendon glide at the site of injury. The skin was closed with interrupted 10/0 polyamide sutures (BBraun Medical, Germany). Mice were euthanized at three weeks and eight weeks following injury. Four hours before tissue collection, each mouse had 10 µl/g of 5-bromo-2-deoxyuridine (BrdU, GE Healthcare, UK) injected into their peritoneal cavity.

Both hindpaws were harvested and immediately fixed, decalcified, tissue processed, serially sectioned at 7 µm and stained with Haematoxylin and Eosin Y (H&E) (Thermo Scientific Raymond Lamb, UK.) and picosirius red (Abcam, UK.) as per manufacturer guidance.

Five sample sections per tendon, 56 µm apart, representing the adhesion and covering 280 µm depth were analysed using Image Pro Plus version 4.5 (Media Cybernetics, USA). From these calibrated images, adhesion length was digitally measured. The area of adhesion and the volume of the deep digital flexor tendon were calculated based on Cavalieri principles [23].

To investigate the remodelling of the tendon architecture, standard histological images were layered onto polarised images for quantification using a modified method from Lin et al [24] (Figure S1). Images of H&E stained histology with bright field microscopy were captured in the same position with the polarising filter (Leica Microsystems, Germany) sited at 45° to the tendon which gave maximum polarisation through aligned collagen. Images were analysed as before and the area of tendon mapped using the outlining function on H&E stained images. The latter image was layered onto the polarised image to generate a precise outline on the polarised image. The quantification counter in Image pro plus, all bright (polarising) areas were quantified as a percentage of the overall tendon area. Six non wounded tendons were also quantified to establish base line levels of polarisation in unwounded tendon. Values measured were tendon volume (µm^3^), adhesion area (µm^2^) and percentage polarisation (%).

### Immunohistochemical Analysis

For analysis of synthetic and proliferative activity between untreated and Adaprev treated tendons 3 representative slides were taken from each serial sectioned digits and antibody stained for 1∶200 (v/v) dilution BrdU (Abcam, UK) and 1∶200 (v/v) dilution heat shock protein 47 (Hsp47; Stressgen, USA), in triplicate per mouse digit. Immunoperoxidase techniques were standardized as previously described [11]. Slides to be stained for Hsp47 antibodies were pretreated with 10 minutes in 4 mol/L HCl followed by 5 minutes in pH 8.2 borate buffer (Sigma- Aldrich, UK.) prior to antibody staining, and a specific mouse on mouse kit (Vector Laboratories, UK) was used. For BrdU antibodies, a standard rabbit anti-rat biotinylated secondary antibody (Vector Laboratories, UK) was used and amplified using the Elite ABC kit (Vector Laboratories, UK). These kits were used as recommended in the manufacturer’s guidelines (Vector Laboratories, UK). Blocking and secondary incubation was performed at room temperature whilst primary incubation was performed at 37°C. Samples were washed twice for five minutes using 0.1% Tween (v/v) in PBS between each step of the protocol. 3,3′diaminobenzidine (DAB) (Vector Laboratories, UK) was used for substrate staining and Nuclear fast red (Sigma- Aldrich, UK.) was used as a counter stain.

In addition flexor tendons in the hindpaws of three C57/BL6 mice were experimentally injured by partial surgical laceration. Lacerated tendons were then treated with either Adaprev or isotonic PBS (control). At days 24 hours after injury animals were euthanized and the tendons recovered and processed for wax embedding as described above.

Immunohistochemical analysis of 7 µm sections was carried out using specific antibodies to visualise the distribution of the M6P receptor (CI-M6PR; Abcam, UK), and the TGF-β receptor 1 (TGF-βR1; Abcam, UK), Smad 2 (Abcam, UK) and Smad 3 (Abcam, UK) which using the rabbit ImmPRESS biotinylated kit (Vector laboratories, UK). Samples were blocked in 2.5% goat serum (Vector Laboratories, UK) for 1 hour at room temperature prior to incubation with each antibody at 1∶200 (v/v) dilution for 1 hour at 37°C. After PBS wash the ImmPRESS kit was applied for 30 minutes, washed and then DAB reacted. Sections were then dehydrated through graded alcohols and transferred to xylene before being mounted on a coverslip. The distribution of these molecules in the treated tendons was compared with that observed in unwounded tissues as controls.

### Rabbit Operative Model

Thirty Adult New Zealand white rabbits (Harlan, UK) were used (average weight of 2.5 kg) and randomized to receive either PBS or Adaprev. Anaesthesia was induced by intramuscular 15 mg/kg Vetalar-V (Pfizer, Surrey, UK) and 0.25 mg/kg Domitor (Pfizer, UK) and maintained with Isoflurane (Abbott, UK), Oxygen and nitrous oxide (BOC Healthcare, UK). Reversal of sedation was performed with Antisedan (Pfizer, UK) 5 mg/ml.

A longitudinal incision was made on the volar surface of the forepaw between the metacarpophalangeal and proximal interphalangeal joints of the middle digit, under 3 times loupe magnification (Surgical Acuity, USA). The flexor sheath was incised. The flexor digitorum profundus tendon was isolated between the A2 and A4 pulleys and sharply transected. An immediate tendon repair was performed with 5-0 Prolene (Ethicon, USA) modified two-strand Kessler repair without an epitendinous suture. 50 µL of either PBS (control) or Adaprev was applied to the tendon repair site and surrounding tissue and allowed to infiltrate for one minute. The skin was reapproximated with a running 4-0 Prolene suture (Ethicon, USA). Chloramphenicol ointment (Abbott, UK) was applied to the wound, and the forepaw was immobilized in an above elbow cast (Lewis-Plast, UK) with the paw and elbow joints in flexion for two weeks.

Ten Rabbits had liquid sample aspirates collected from the intra-synovial junction of the treated tendon sheath at 0, 5, 15, 30 and 45 minutes and diluted in water (1∶100) followed by a 1∶25 dilution in PBS to attain a suitable concentration for high performance anion exchange with pulsed amperometric detection (HPAE-PAD) quantification using a Dionex ICS-5000 (Thermo Scientific, Germany) 20 µL of the diluted sample was injected on a strong anion exchange column designed for selective carbohydrate separations. M6P is eluted using a gradient of 47.5 mM sodium hydroxide (Life Technologies, USA) and 500 mM sodium acetate at 1 mL/min over 20 min, and detected using a Four-Potential Waveform.

The remaining 20 rabbits were studied at six weeks postoperatively (n = 10 in each group), the animals were killed and the left forepaw of each rabbit was removed. Tendon’s were harvested and then tested in an Instron 5542 Tensiometer System (Instron, USA) controlled by Bluehill2 software (v2.9). Tendons were loaded longitudinally along the axis of the fibers and distracted at 20 mm/min, using 500N load cell (Instron, USA), as this gave most reproducible data. Force and extension data were recorded every 100 ms. Young's modulus, ultimate load to failure, Max force and force normalised for cross sectional area were calculated.

### Cell viability assay

Freshly harvested C57/Bl mice flexor tendons were digested in collagenase I (2.5 mg/mL, Sigma-Aldrich, UK) for 3 hours at 37°C, pipetting gently every 30 minutes. Digests were then centrifuged at 300 g for 15 minutes and resuspended in Dulbecco’s Modified Eagle Media (DMEM; Life Technologies, UK) with 10% fetal bovine serum (FBS; Life Technologies, UK). These were grown to confluence for 5 passages and seeded at approximately 20,000 cells per well in a 96 well imaging plate (BD Biosciences, USA). Wells were rinsed with PBS prior to drug treatment with 50 mM, 200 mM or 600 mM M6P (Adaprev) for 45, 60, or 120 minutes. Control wells were treated with DMEM only or 95% methanol (Sigma-Aldrich, UK.) for the corresponding times. After the specified time, wells were washed with PBS and incubated for 30 minutes with 4 µM Ethidium homodimer-1 (EtHD-1) and 2 µM Calcein AM (Molecular Probes LIVE/DEAD viability/cytotoxicity kit, Life Technologies, USA). All treatments were performed in triplicates. Images of cell viability were acquired on a Pathway Bioimager 855 (BD Biosciences, USA) and the following filter setup: Ex. 360/10, FITC 488/10 and 555/28; Em. 84101(Quad). Images were collected in each well with an offset from the well centre of 10×10 µm and a montage of [3×3] was created without gaps. Exposure times for each fluorophore were calculated automatically and threshold masks were applied to each image using the automatic feature of the software. The images were then processed and analysed in ImageJ software. An intensity threshold of >500 for Calcein AM (live) and >2000 for EtHD-1 (dead) was applied for each channel and the number of live and dead cells was quantified using the Analyse Particles module (BD Biosciences, USA). Stress-shielded cells were quantified manually based on their shape, defined as cells without any cytoplasmic protrusions, exhibiting a condensed round morphology using ImageJ [25].

### Rat tendon fibroblast culture

Flexor tendons from Male Sprague-Dawley rat hindpaws (Harlan, UK) were dissected out and placed into L15 air-buffered culture medium (Sigma-Aldrich, UK) and minced into 5 mm tissue pieces and seeded into a Petri dish (Corning, USA). After addition of growth medium (DMEM/10% Foetal Bovine Serum (FBS) (v/v), tissue was then incubated for 3days to allow fibroblast outgrowth until cells were 80% confluent.

### p38 Immunoblot

Rat hindpaw flexor tendon fibroblasts were seeded at 300,000 cells per well of a 6 well plate (Nunc, Thermo scientific, USA), in DMEM supplemented with 10% (v/v) FBS, 0.1 mM non-essential amino acids, 2 mM glutamax (Life Technologies, USA) and 100U penicillin/100 µg streptomycin (Life Technologies, USA). After one day the cells were serum starved in DMEM supplemented with 0.1 mM non essential amino acids (Life Technologies, USA), 2 mM glutamax and 100 U penicillin/100 µg streptomycin. The cells were then treated with Adaprev or 600 mM G6P for 5, 10, 15, 30 and 60 minutes with untreated cells as a control. All treatments were performed in triplicate. After treatment the cells were lysed in lysis buffer (Urea [8M], (Life Technologies, USA); Thiourea [1M], (Sigma-Aldrich, UK), CHAPS [4% w/v] (Sigma-Aldrich, UK), Dithiothreitol [65 mM], (MP Biomedical, USA) Protease Inhibitor [0.5% w/v] (Roche, Swtizerland); Phosphatase Inhibitor 1 [1% w/v], (Sigma-Aldrich, UK) Phosphatase Inhibitor 2 [1% w/v], (Sigma-Aldrich, UK)). Samples were then subjected to gel electrophoresis, transferred onto nitrocellulose and immunoblotted with a phospho-p38 antibody (Cell signalling technologies, USA).

### F-actin cytoskeleton rearrangement

Rat hindpaw flexor tendon fibroblasts were seeded in Nunc Labtek 8-well chambers at 23×10^3^ cells/mL (0.3 mL/well) (Thermo scientific, USA), in DMEM/10% (v/v) FBS, and incubated overnight at 37°C/5% CO_2_. Following incubation, cells were treated with Adaprev, fixed in 4% formaldehyde for 10 minutes and washed 3×5 minutes in PBS. Cells were permeabilized in 0.25% (v/v) TRITON-X/PBS (Sigma-Aldrich, UK) for 10 minutes, followed by brief PBS washes, incubated with 1%(w/v) BSA/PBS (Sigma-Aldrich, UK) for 30 minutes to block non-specific antibody binding. Finally, cells were briefly washed in PBS and incubated with Alexafluor phalloidin (1∶40 dilution) (Molecular Probes, USA), in 1%(w/v) BSA/PBS, washed in PBS and incubated for 10 minutes at room temperature with DAPI (1∶100 dilution) (Molecular Probes, USA). All treatments were performed in triplicate. Cells were washed in PBS and were mounted in Vectashield (Vector laboratories, UK) under a coverslip and visualised with a Leica DM6000B fluorescent microscope (Leica Microsystems, Germany) and captured using Leica Application Suite 2.1.0 (Leica Microsystems, Germany).

### Cell Migration Assay (Chemokinesis)

Rat hindpaw flexor tendon fibroblasts in DMEM/10% (v/v) FBS were seeded into a Corning Costar 24-well plate (Sigma-Aldrich, UK) at 2.1×10^4^ cells/well and incubated overnight. Cells were treated with Adaprev or 600 mM G6P, washed in sterile PBS and incubated in DMEM/10%FBS and the movement of 10 fibroblasts per treatment group were assessed using a Leica DMIRB Inverted timelapse video microscope with phase contrast (Leica Microsystems, Germany). Control cells were exposed to DMEM/10%FBS (v/v) (positive control) or DMEM alone (negative control). Timelapse images were taken every 15 minutes for 20 hours, at 7.5 times magnification and were imported into ImageJ software. where cell motility in each treatment was recorded using the Manual Tracking plugin. Motility data were imported into the Chemotaxis and Migration Tool plugin (Ibidi, Germany) to generate plots of migration paths and distances from a normalised point of origin. Further comparisons between treatment groups were made determining the percentage of cells migrating pre-determined distances.

### Cell Migration Assay (Chemotaxis)

Rat hindpaw flexor tendon fibroblasts were seeded onto a Corning 96-well transwell filter (pore size 8 µm) (Corning, USA), at 5×10^4^ cells/filter. DMEM/10% (v/v) FBS was added into the lower chamber. Cells were incubated overnight to allow adhesion to the filter. Following incubation, medium was aspirated from both chambers and filters washed with sterile PBS before incubating the cells in Adaprev or 600 mM Glucose-6-Phosphate (G6P) added to both chambers of the transwell, for 0, 15, 30, 45, 60 and 120 minutes. After treatments were performed in triplicate wells, cells were washed in PBS and cell migration initiated by adding DMEM/10% (v/v) FBS to the lower chamber of the transwell, and serum-free DMEM to the upper chamber. Following overnight incubation, well inserts were treated with Accutase (Sigma-Aldrich, UK) for 5 minutes to remove migrated cells from the underside of the filter into a receiver plate. Cell Titre Glo/10% (v/v) FBS (Promega, UK) was added to the migrated cells which were shaken for 2 minutes, incubated at room temperature for 10 minutes, and counted using a 340PC384 SpectraMax plate reader (Molecular Devices, USA).

### Cell Proliferation Assay

Rat hindpaw flexor tendon fibroblasts were plated out at 10^4^ cells per well in a 96 well plate (Corning, USA), and incubated overnight at 37°C. Cells were exposed to Adaprev or G6P (600 mM), in triplicates, for 15, 30, 45, 60 and 120 minutes. Following exposure, treatments were removed and all wells washed twice with PBS, and wells were treated with growth medium (DMEM/10% FBS (v/v). Cells without Adaprev or G6P treatment acted as positive controls. Cell proliferation was determined using a cell counting kit (CCK-8, Fluka, Sigma-Aldrich, UK) at 12, 24, 48, 120, 144, 168 and 192 hours post treatment. The percentage cell proliferation relative to the positive control (DMEM/10%FBS (v/v) with no treatment) for triplicate wells was determined.

### *Ex Vivo* Cell Migration and Proliferation

Flexor tendons from six rat hindpaws were dissected out and placed into L15 air-buffered medium. Tendons were oriented longitudinally in rat collagen-I matrix (BD Biosciences, USA). DMEM/10% (v/v) FBS was added and the tendons were incubated for 48 hours. Medium was then removed and replaced with Adaprev for one hour. After the 1 hour incubation the treatment was replaced with DMEM/10%FBS (v/v). Tendons exposed to DMEM/10%FBS (v/v) alone acted as positive controls. Tendons were photographed every 24 hours to assess and compare fibroblast outgrowth from tendons.

### Statistical Analyses

All experiments were analysed in a blinded manner by at least two independent observers and sampled accordingly to generate mean values expressed with standard error of mean (±). Between mouse *in vivo* replicates, treatments were analysed for differences between groups using paired Student’s t-test based on the null hypothesis of no difference between active drug treatment and control. Between rabbit in vivo experiments, treatments were analysed between groups using independent Student’s t-test based on the null hypothesis of no difference between active drug treatment and control. In culture experiments were performed in at least triplicate and comparisons were made using one-way ANOVA between treatments using statistical software (IBM SPSS Statistics v.20). A p value of less than 0.05 was considered to be significant.

## Results

### Intrathecal administration of Adaprev reduces *in vivo* tendon adhesion formation

Within 24 hours of injecting DiI, the dye was widely distributed within the tendon sheath (Figure S2) coating the entire length of the tendon and sheath within the digit. Treatment of tendons with TGF-β1 increased adhesion formation by 149% and 46% at three and eight weeks respectively but not found to be statistically significant.

**Figure 1:**
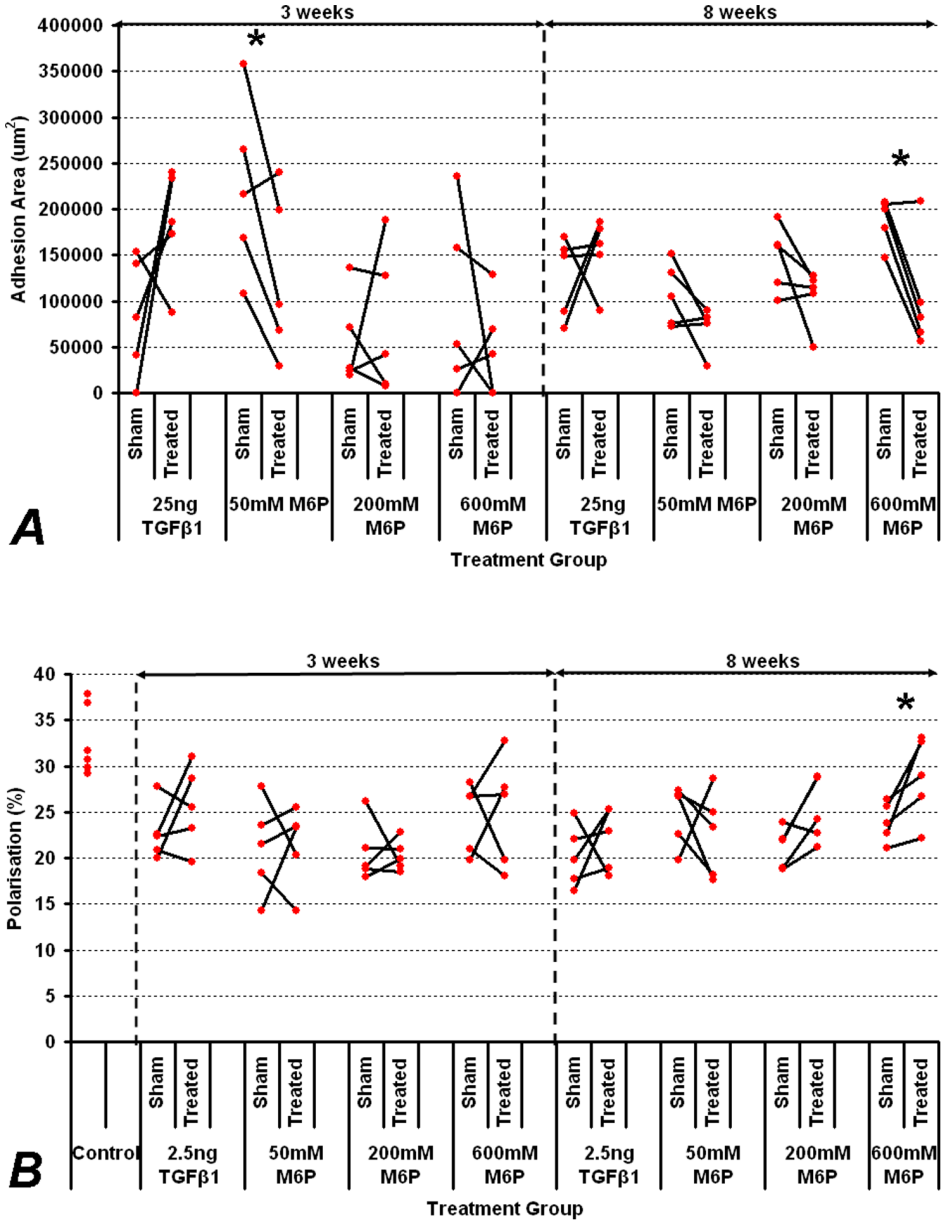
Effect of M6P on tendon adhesion area and tendon structural organisation. A. Effect of M6P at increasing doses on adhesion area at 3 weeks (most synthetic period) and 8 weeks (end of tendon healing). B. Effect of M6P at increasing doses on tendon organisation as measured by quantitative polarisation. Effect of TGFβ-1 at 2.5 ng was used as a positive control but did not significantly increase adhesion formation or improve tendon organisation (p>0.05). All values of treated tendons were compared to contralateral saline controls. Unwounded controls were also measured for polarisation normalisation. Significant reductions in adhesion formation were seen in 50 mM M6P treated group at three weeks and in 600 mM M6P treated groups at eight weeks. Improvement in tendon organisation was significant with 600 mM M6P treatment. Significant differences with p<0.05 on paired t-testing are indicated by an asterisk.

**Figure 2:**
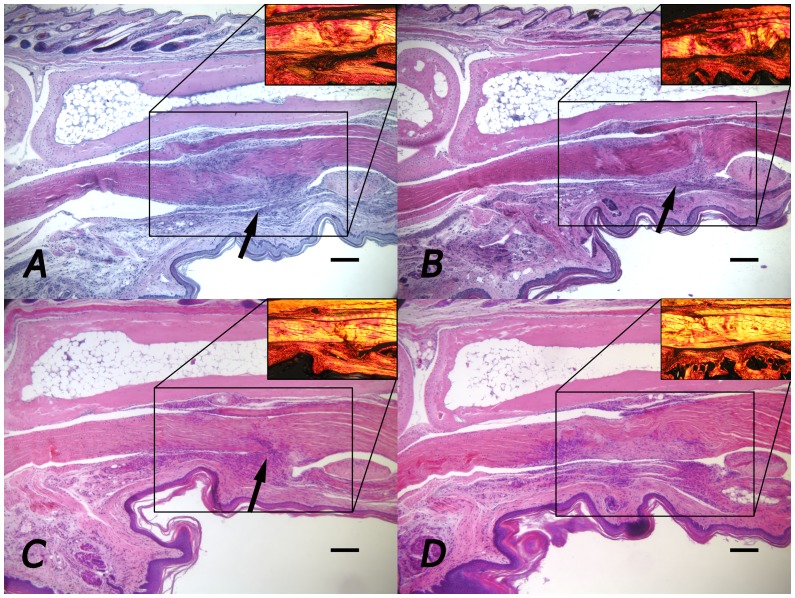
Representative histological images of stained flexor tendon adhesions. A. Three week sham treated tendon. B. Three week 600 mM M6P treated tendon C. Eight week sham treated tendon. D. Eight week 600 mM M6P treated tendon. Arrows indicate site of adhesion. Sub image shows area stained with picosirius red. Note majority of fibres seen type I collagen (red/yellow) with very little type III collagen (green). Adhesion made up of less densely packed type I collagen fibres. Note sham treated tendon appears more cellular and has a larger area of adhesion than tendon treated with 600 mM M6P. No adhesion in D. 600 mM M6P treated tendon. Scale bar represents 200 µm.

Treatment with 50 mM and 200 mM M6P had no significant effect on adhesion formation at three or eight weeks however, the Adaprev treatment group displayed a reduction in tendon adhesion formation three weeks post-surgery (mean reduction in adhesion area of 39% compared to contralateral controls) although this improvement was not significant. At eight weeks post-surgery, a significant reduction in tendon adhesion formation was observed compared to the saline control with Adaprev treatment (p = 0.02) with a mean percentage reduction in adhesion area of 47% (Figure 1A). In this group there was also a significant improvement in tendon organisation as measured by quantitative polarisation (87.7% normalised treated compared to 32% normalised controls, p = 0.047) (Figure 1B). Histologically, notable differences in the amount of adhesion that formed were evident in 600 mM treated tendons when comparing paired samples at both three and eight weeks (Figure 2). Staining with picosirius red at 3 and 8 weeks showed less densely packed type I collagen fibres (red/yellow) at the adhesion site with little evidence of type III collagen (green). Collagen type I fibres were most evident throughout the tendon with no discernable difference was detectable between Adaprev and untreated groups at either 3 or 8 weeks (Figure 2).

**Figure 3:**
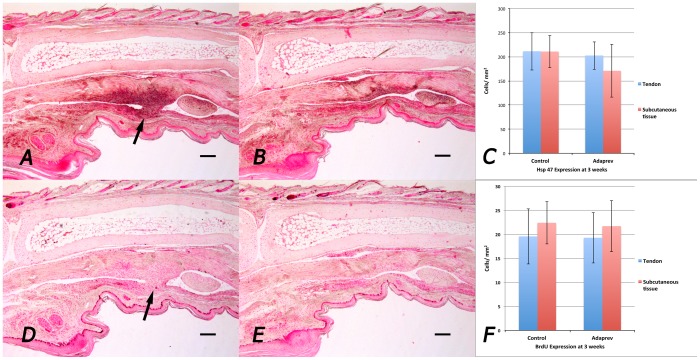
*In vivo* collagen synthesis and cell proliferation by immunohistochemistry. Representative samples were taken from 3 week injured flexor tendons. A. Sham treated flexor tendon injury stained with antibodies to Hsp47 (collagen synthesis marker). Arrow indicates adhesion. B Adaprev treated flexor tendon injury. C Quantitative analysis of Hsp47 in tendon and subcutaneous tissues showing no significant difference in cellular Hsp47 expression between the two groups. D. Sham treated flexor tendon injury stained with antibodies to BrdU (cellular proliferation marker). Arrow indicates adhesion. Note good staining of basal layer of skin, hair follicles and on the tendon surface. E. Adaprev treated flexor tendon injury. F. Quantitative analysis of if BrdU in tendon and subcutaneous tissues showing no significant difference in cellular BrdU expression between the two groups (p>0.05). Error bars represent standard error of mean. Scale bar represents 200 µm.

Staining for Hsp 47 (marker for collagen synthesis) at 3 weeks as the point of maximal cellular activity showed increased Hsp 47 expression at the site of skin wound, tendon wound and if present, adhesion but showed no significant difference between untreated and Adaprev treated tendons (211±39 cells/mm^2^ vs 202±29 cells/mm^2^ respectively; p>0.05; (Figure 3 ABC). Likewise staining for cellular proliferation (BrdU) showed no difference no significant difference between untreated and Adaprev treated tendons at 3 weeks (20±6 cells/mm^2^ vs 19±5 cells/mm^2^ respectively; p>0.05; (Figure 3 DEF).

### TGF-β pathway receptors and downstream target expression are absent 24 hours after injury

Immunostaining for CI-M6PR, TGFβ -R1, SMAD 2 and SMAD 3 revealed no expression of these receptors in the first 24 hours after injury, which is beyond the expected residency time of M6P despite positive staining in unwounded controls (Figure S3).

### Residency of Adaprev in the flexor sheath is short

Analysis of the biological availability of Adaprev *in vivo* showed that over 45 mins there was a significant reduction of bioavailable M6P in the flexor sheath by 40% (Figure S4).

### Treatment with Adaprev did not weaken tendon repairs

**Figure 4:**
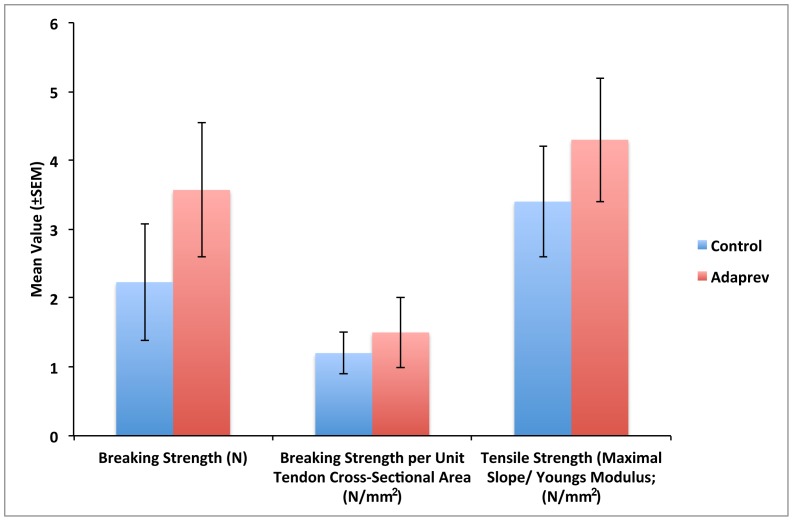
Effect of Adaprev on Rabbit flexor tendon breaking strength *in vivo*. No significant difference in breaking strength and tensile strength between treated and non treated flexor tendons (p>0.05). Error bars represent standard error of mean.

Tendons repaired using a standard modified two core Kessler repair treated with Adaprev did not demonstrate an increased predisposition to rupture with breaking strengths repair greater than controls (3.57 N c.f 2.23 N) however this was not statistically significant (p>0.05). When normalised for tendon cross sectional area both breaking strength and tensile strength showed no significant difference between Adaprev and no treated controls (p>0.05) (Figure 4).

Based on this data 600 mM M6P (Adaprev) was selected as the most therapeutically active concentration to reduce adhesion formation without apparent detriment to collagen synthesis or cellular proliferation at the peak stages of tendon healing and therefore used to further investigate mechanism of action.

### Adaprev was not cytotoxic and induced features of cell stress

**Figure 5:**
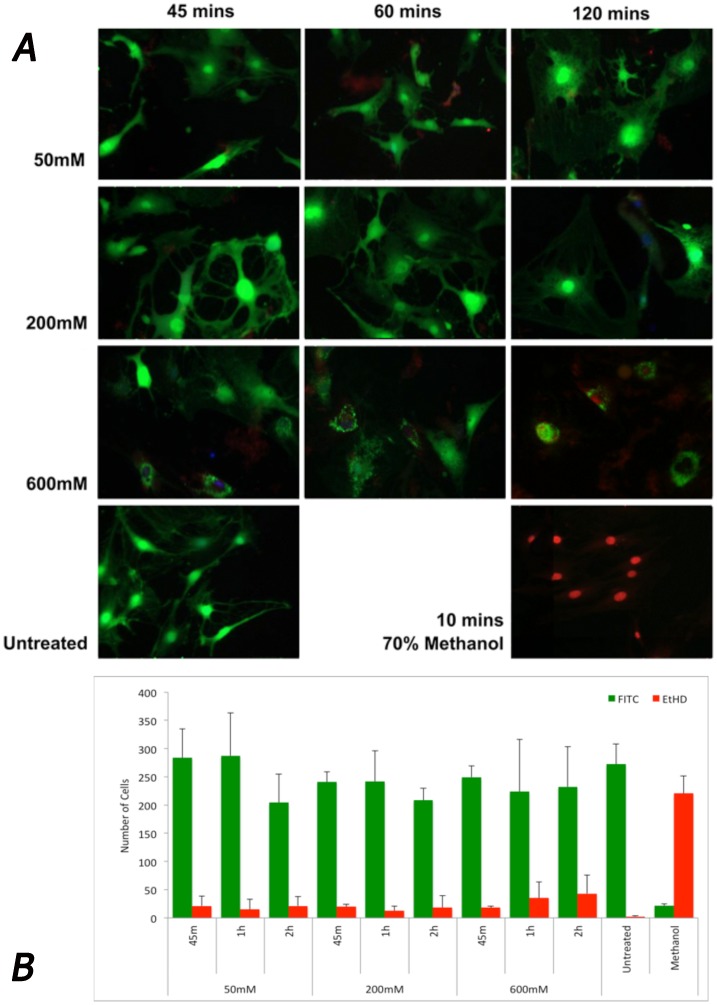
Cell viability following increasing doses of M6P treatment. A. Live/Dead viability stain used to exhibit cell morphology and cell viability. Living cells uptake calcein AM (green) Ethidium homodimer taken up by dead cells (red). Untreated cells show spindle shaped morphology in confluence whereas treated cells with 50 mM retain their spindle morphology at 45 and 60 minutes of exposure. At 200 mM cells begin to lose their spindle morphology but continue to have cytoplasmic protrusions with evidence of crenation after 120 minutes of exposure. Cells treated with 600 mM showed fewer cytoplasmic protrusions with a considerable shielded appearance after 60 minutes and 2 hours. B. Quantification of the living (green) and dead (red) cells revealed the majority of cells were still viable after all treatments with no significant loss of cellular viability (p>0.05). No significant difference was found between the number of live cells or dead cells found between treatments, dosages or exposure times, except those observed in the negative control (methanol) (p>0.05). Error bars represent standard error of mean.

Tendon fibroblasts in culture developed a spindle shaped morphology in culture but once exposed to increasing doses of M6P developed increasingly rounder morphologies with all cells viable (Figure 5 A). The number of completely rounded cells was quantified (no cytoplasmic protrusions) and shown to present mostly in the 600 mM M6P treated group at increasing numbers the longer the cells were exposed (Figure 5B). The number of cells that was stress-shielded was counted and reported here as a percentage of the total cells observed (Figure S5). We found that after 45 mins of 600 mM M6P exposure, just over half (54%) of cells were stress-shielded, which was significantly more than compared cells exposed to 200 mM (2.9%, p = 0.038). Indeed, only 2.3% of cells were found not to be stress-shielded after 2 hours at 600 mM exposure. There was no significant increase in cell death as measured by ethidium homodimer uptake with increasing concentration or duration of exposure to M6P (p>0.05).

### Increased concentration of M6P related directly to increased osmolality

We were surprised by the high number of stress-shielded cells so we measured the osmolality of the solutions of M6P. We found a linear relationship with the concentration of M6P (Figure S6) and the osmolality. 600 mM M6P (Adaprev) was the highest concentration we could reliably reproduce and was considerably hypertonic at 1500 mOsm, as was 200 mM M6P at 689 mOsm and to a lesser extent 50 mM M6P at 395 mOsm. We hypothesised that high osmolar application of M6P may have biological effects *via* osmotic shock and therefore we compared Glucose 6-Phosphate (G6P), a similar sized sugar molecule not involved in the TGF-β pathway, to see if we could replicate this effect.

### Adaprev has comparable p38 induction as G6P

**Figure 6:**
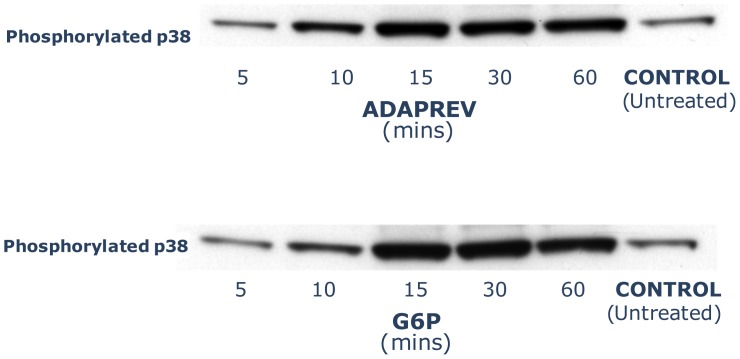
Phosphorylation of p38 by Adaprev and G6P. Both Adaprev and G6P significantly increased phosphorylation of p38 when compared with controls with peak activity between 15 and 60 minutes of exposure.

G6P is a monosaccharide that has similar physical properties and same molecular weight as M6P (282.12; and same tonicity in solution), but has a low binding affinity for the CI-M6PR [26] and therefore has no significant effects in CI-M6PR and little pharmacological activity (Figure S7). Expression of phosphorylated p38 was induced by both hypertonic 600 mM G6P and Adaprev with maximal induction at 15 to 60 minutes (Figure 6) to a far greater extent than the DMEM/10% FBS (v/v) controls.

### Adaprev treatment affects cytoskeletal organisation similar to G6P

**Figure 7:**
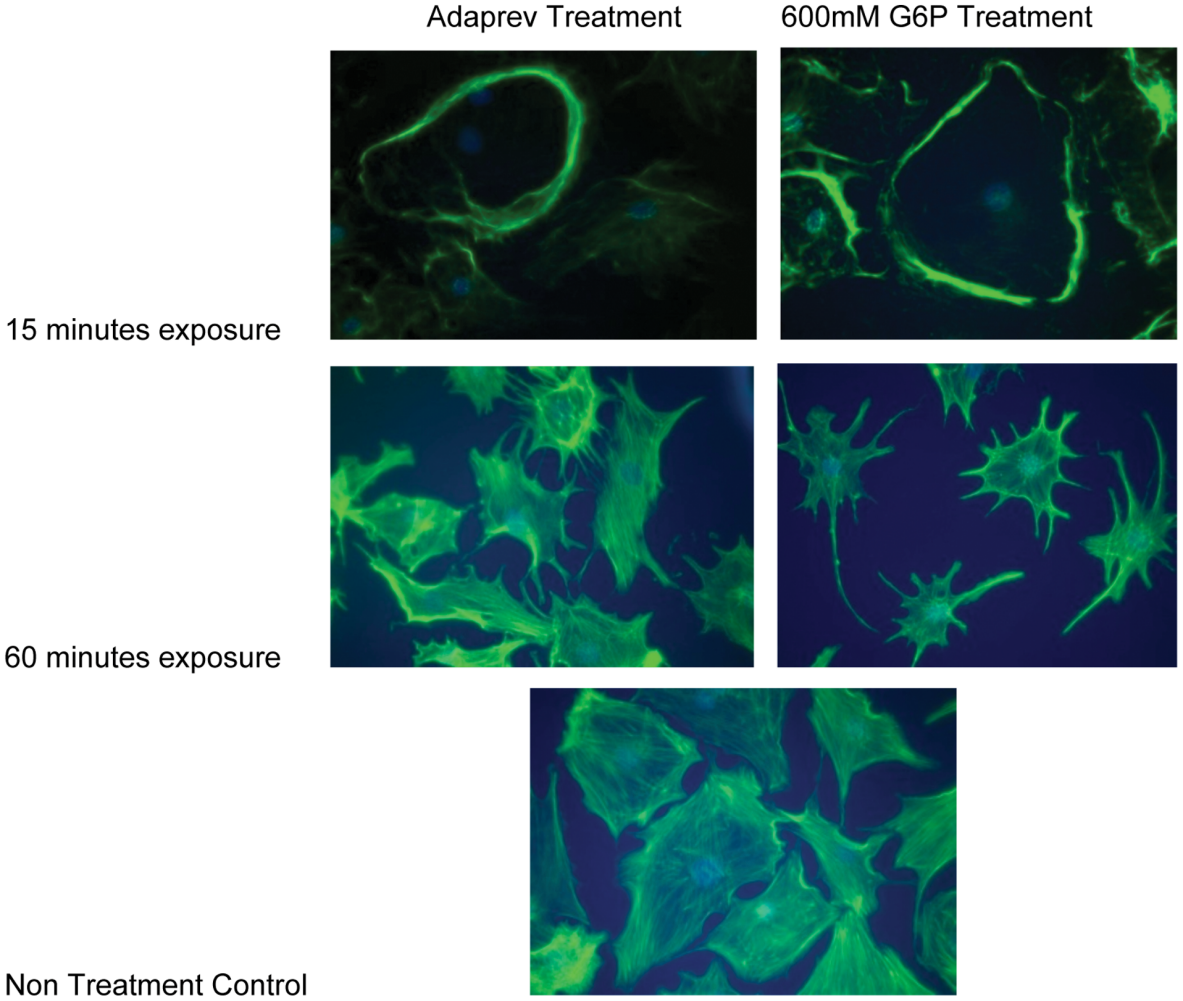
Effect of Adaprev and G6P on actin cytoskelaton of rat flexor tendon fibroblasts. Rat flexor tendons were grown in DMEM containing 10% Fetal Bovine Serum (FBS) and then transferred to media without FBS before either having Adaprev or 600 mM G6P added to the media. Non treatment controls were maintained in DMEM without serum. Cells were maintained under the above conditions for up to 60 minutes and photographed at various times to record the cellular effects. At 15 minutes the cells in both groups show “stress shielding” of the actin cytoskeleton then at 60 minutes similar crenation effects upon both the Adaprev and G6P treated cells were observed.

Adaprev treatment of tendon fibroblasts leads to reversible actin cytoskeletal reorganisation compared to *in vitro* FBS controls (Figure 7). Adaprev treatment resulted in a relatively rapid movement of actin fibres to the cell periphery (within 15 minutes of exposure), a classical osmotic response triggered by the cell to maintain its shape (stress-shielding). In the following 30–60 minutes after Adaprev exposure cells began to show signs of crenation with the actin cytoskeleton forming a network around the nucleus and losing its spindle shaped morphology. This ‘stressed’ appearance persisted until subsequent dilution of Adaprev with media changes. Comparable results were observed with 600 mM G6P indicative of osmosis being a colligative property.

### Adaprev inhibits fibroblast migration

**Figure 8:**
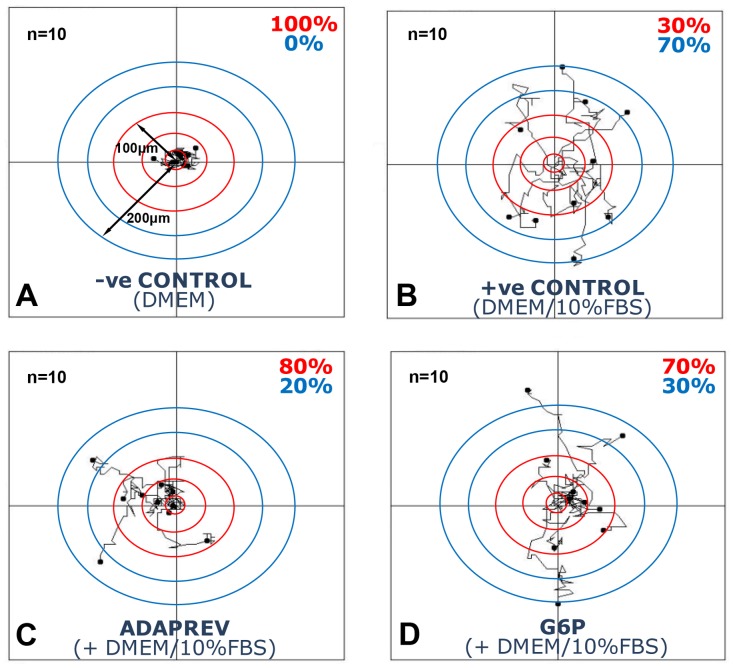
Effect of Adaprev on fibroblast migration (Chemokinesis). A. Control without 10% FBS. B. Control with 10% FBS. C. M6P with 10% FBS. D. G6P with 10% FBS. Concentric rings indicate 50 µm distance from centre with ten cells measured per treatment group. Note 100% of cells within 100 µm distance with no FBS. Note 70% of cells had migrated beyond 100 µm in DMEM and 10% FBS controls. Adaprev and G6P treatment resulted in a reduction of migration with only 20% and 30% of cells migrating beyond 100 µm.

The addition of 10% FBS significantly increased cell movement in a random walk pattern (Chemokinesis) compared with DMEM only controls with the mean walk distance for 10 mapped cells 278.2±23.32 µm over 20 hours. Following treatment with Adaprev, cell migration was reduced significantly to a mean of 143.1±29.9 µm (p<0.05) (Figure S8). G6P also reduced cell migration compared to DMEM/10%FBS (v/v) controls but this was not significant (202.3±34.5 µm). Comparing cell migration away from central 50 µm concentric rings showed that control tendon fibroblasts cultured in DMEM only solution demonstrated 100% of cells within a 100 µm radius (Figure 8A). Seventy percent of tendon fibroblasts cultured in DMEM/10%FBS (v/v) migrated beyond 100 µm (Figure 8B). Tendon fibroblasts treated with Adaprev however showed only 20% of cells migrated beyond 100 µm (Figure 8C) and those treated with G6P found 30% migrated beyond 100 µm (Figure 8D).

Transwell plate migration studies (Chemotaxis) found that the duration of exposure to Adaprev or G6P had a profound effect on migration through the transwell plate. Increasing duration of Adaprev exposure significantly reduced the luminescence from cell reader by 58% at 15 minutes exposure, 63% at 30 minutes, 91% at 45 minutes, 92% at 60 minutes and >99% at 120 minutes (p<0.05). G6P also reduced migratory capacity of fibroblasts but to a lesser extent by 17% at 15 minutes exposure, 30% at 30 minutes, 44% at 45 minutes, 69% at 60 minutes and >99% at 120 minutes (p<0.05) (Figure S9).

### Adaprev delayed onset of proliferation on cultured fibroblasts

**Figure 9:**
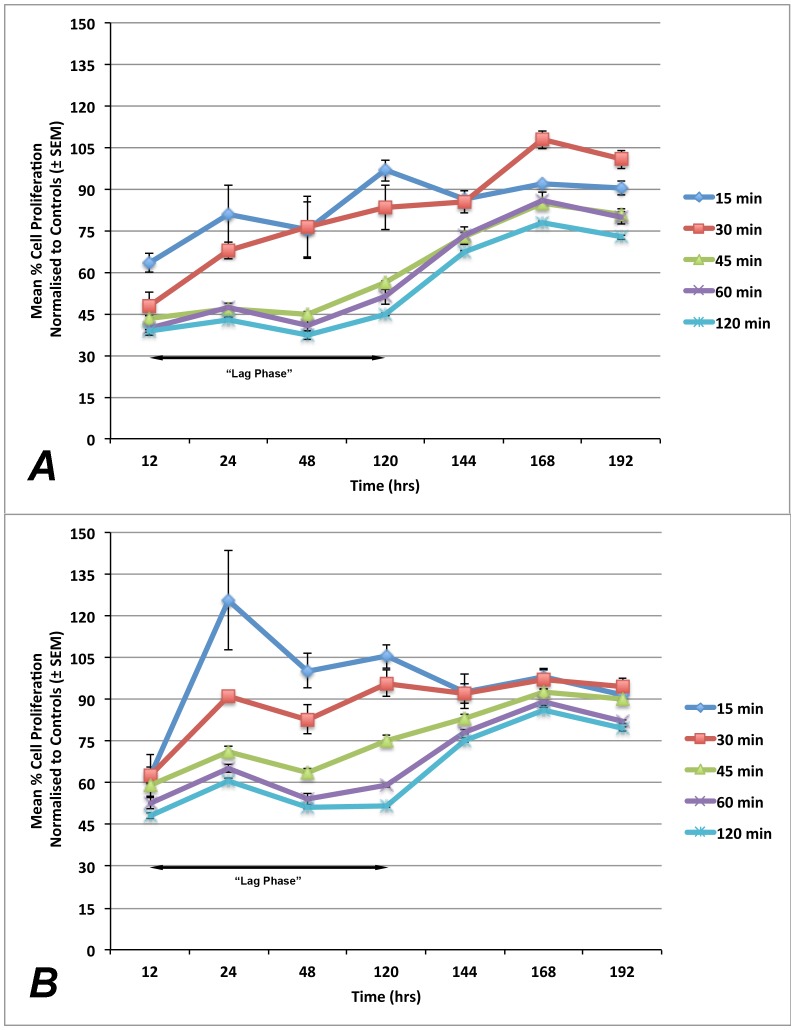
Effect of Adaprev exposure time on fibroblast proliferation. Proliferation activity was normalised to untreated cells and treated cells expressed as a percentage of non-template controls. A. Exposure to Adaprev significantly reduced cell proliferation (p<0.05), which gradually recovered to normal levels in 15 minutes and 30 minutes exposure groups after 120 hours. Longer periods of exposure (45–120 minutes) showed a significant “lag phase” which gradually recovered to near normal levels at 168 hours B. Treatment with G6P showed similar “lag phase” kinetics. Error bars represent standard error of mean.

Fibroblast proliferation was compared to non-treatment controls and found that both Adaprev and G6P had a temporary inhibitory effect on cell proliferation at increasing levels of exposure. This demonstrated a significant “lag phase” compared to normal which for short exposure (15–30 minutes) recovered by 120 hours but with longer exposures (45–120 minutes) recovered slowly after 168 hours (p<0.05) (Figure 7). The effect of short exposure of 15 minutes and long exposure of 120 minutes was found to be significantly different (p<0.05). The effect of duration of Adaprev exposure on cell proliferation was investigated and showed that after 15 and 30 minutes exposure to Adaprev *in vitro*, little effect on cell proliferation was observed. Increasing exposure time of the cultured fibroblasts to Adaprev for 45, 60 and 120 minutes resulted in a prolonged “lag phase” of proliferation of four to five days before cell proliferation began to return to normal levels (Figure 9).

### Adaprev delayed migration and proliferation of fibroblasts from ex vivo model

**Figure 10:**
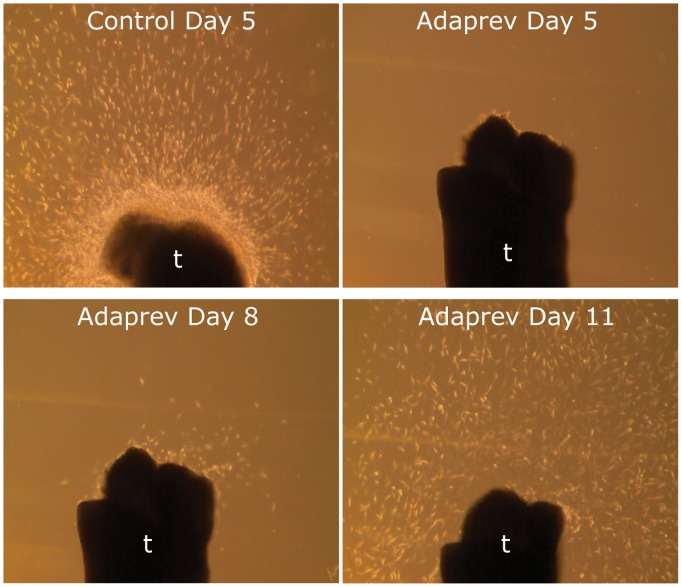
*Ex vivo* migration assay. Control cut tendon ends demonstrate a vast number of cells migrating from the tendon after 5 days in DMEM/10%FBS culture medium. Tendon treated in high Adaprev only began showing signs of cell migration at 8 days which gradually normalised after 11 days.

The “lag phase” seen in the proliferation studies and reduction of cell migration effect of Adaprev was mirrored in the *ex vivo* whole mount tendon studies. In untreated tendon in DMEM/10%FBS (v/v) significant outgrowth was seen at 5 days however after exposure to Adaprev for 1 hour, cells remained within the tendon, with migration from the tendon ends initiating at approximately 8 days following treatment with only a normalising pattern migration occurring at 11 days (Figure 10).

## Discussion

The estimated direct cost to healthcare of a poor functioning finger after flexor tendon injury is approximately $7000, with indirect costs to society through loss of earnings or workforce $13200 [27]. There are few effective treatments against tendon adhesion formation hence potential therapies to combat adhesions could have a significant healthcare impact. Numerous therapies have been investigated in order to determine their efficacy in reducing tendon adhesions and few if any achieve clinical application [9].

Many studies have shown that M6P reduces tendon adhesions by antagonism of the TGF-β pathway and proposed the mechanism of action is via suppression of latent TGF-β activation [14], [20], [28]. M6P is a low molecular weight monosaccharide that competitively binds to CI-M6P receptors, which are required to activate latent TGF-β1 receptors hence reducing locally available active TGF-β1 [29]. The proposed mechanisms by which latent TGF-β is activated include formation of a CI-M6PR complex with urokinase plasminogen activator receptor (uPAR) which in turn converts plasminogen to plasmin which in turn activates TGF-β1 [30]. A number of studies have subsequently put this to question such as Barnes et al. (2012) [18] who have shown that latency associated peptide of TGF-β1 is not subject to mannose phosphorylation, hence the addition of M6P has little to no effect on inhibiting activation of this peptide. To further complicate these observations it has been shown that CI M6PR may or may not activate latent TGF beta depending on cell type [31]. However the amount of latent TGF beta bound to the extracellular matrix and liberated after injury is likely to be profound [32] and inhibiting its activity by a short-lived peptide would be difficult to achieve.

In this study a 600 mM dose of M6P (Adaprev), significantly caused a 47% reduction in tendon adhesion and a 20% improvement in collagen alignment at eight weeks post-wounding for tendon when compared with contralateral controls (p<0.05). In addition we found little to no effect on collagen synthesis or cell proliferation at the crucial stages of tendon healing and collagen architecture showed predominantly normal levels of collagen type I fibres with the only real difference being the reduction of adhesions and improvement of organisation of collagen in Adaprev treated groups. Importantly the treatment of tendons using Adaprev did not impair the breaking strength of the tendon and therefore could be used as a safe treatment for the use in the clinical setting. This is particular important as previous applications of anti-adhesion therapies such as Adcon T [33] were withdrawn from clinical use after they were found to increase rupture rates in clinical trials.

Our study did not show CI-M6PR, TGFβ-R1 and downstream targets such as SMAD 2 and 3 expression in the first 24 hours of tendon injury in our mouse model suggesting bioavailable M6P did not mediate its effect through the described TGF-β pathway. The effect of altering the concentration of M6P was not cytotoxic to cells even at high doses (600 mM M6P) but did appear to have profound effect on cell morphology. This prompted us to explore the osmolality of M6P, which highlighted that concentrations of 50 mM, 200 mM and 600 mM were 395 mOsm, 689 mOsm and 1500 mOsm respectively. We were surprised to find that this osmolality of sugar did not cause a dramatic loss of cell viability especially as lesser concentration (500 and 700 mOSm) of sucrose have shown to induce cell death in odontoblast cell lines [34]. However the bioavailability of M6P had already reduced by 40% in 45 minutes in our study and as the half-life of M6P is less than 120 minutes *in vivo* [35], it appears that this is sufficiently short that the cells recover. In addition tendon fibroblasts may be particular resistant to the osmotic forces as they regularly tolerate physical stresses from compression, tension [36] and heat [37]. As such the possibility of osmotic shock as a potential mechanism for the biological changes arose.

Cellular responses to hyperosmotic stresses are well described following exposure to high sodium chloride levels or high urea levels [38] and exposure to simple sugars such as sorbital and G6P. Cultured tendon fibroblasts following exposure to hyperosmolar M6P show rapid actin stress fibre reorganization, results which were similar to those seen of Swiss 3T3 cells exposed to 0.45M sucrose [39].

Hyperosmolar G6P, which has a similar molecular weight, tonicity and composition as M6P, was used as a positive control for investigating the osmotic shock potential of Adaprev by comparing phosphorylation of p38 in treated fibroblasts. This is a well established mitogen activated protein kinase pathway for a number of causes of cellular stress [40] however it is particularly sensitive for osmotic stress [41], [42] and hence chosen to be investigated. The increased phosphorylation of p38 in the absence of inflammation, cell migration and proliferation [43] would certainly suggest its association with osmotic shock. Indeed the reconfiguration of the actin cytoskeleton to stress-shielding along the periphery and crenation are characteristic signs of a cells response to hypertonicity [44]. These findings supported by the reduction of cell migration (both chemokinesis and chemotaxis) and cause of a “lag phase” in cell proliferation in both *in vitro* and *ex vivo* models are certainly indicators that the normal cellular wound healing processes are disrupted. Small changes in cell volume caused by hyperosmotic stress can influence cellular migration and proliferation [45] however the cells can adapt to these changes [46]. Studies have shown that osmotic stress can inhibit proteasome function that is key to a number of biological processes via p38 MAPK phosphorylation [47], which may involve cell cycle arrest [48].

The inhibition of both migration and proliferation without apparent effect on collagen synthesis at critical periods of tendon healing (3 weeks) may actually be crucial for the effects of reducing tendon adhesions. The cells lie stationary in their resident tissue and produce collagen without seeding collagen along a proposed migratory path. Indeed the effect of 5-Fluorouracil, a chemotherapeutic drug that has long been reported as having anti-adhesion properties exerts its effect by reducing cell mitosis and inhibiting cell migration [49], [50]. This potential mechanism of action, although simplistic, is evident in the results shown and may actually be of value for developing other treatments for conditions that involve healing between two gliding tissues.

Other markers to consolidate the role of osmotic stress such as TonEBP [51] would also be important to investigate however not within the scope of this study. Future studies will aim to develop the pharmacological role of osmotic stress as a potential new direction to reduce tendon adhesions without any detriment to cell viability and healing. The applications could involve treatments for peritoneal, tendon and corneal adhesions, effectively any conditions where scarring occurs between two surfaces restricting glide.

Taken together the data presented here demonstrate using *in vitro, ex vivo* and *in vivo* experiments that Adaprev appears to have a mode of action independent of the TGF-β pathway, possibly via a physical, hyperosmotic effect. At the cellular level evidence of reversible cell crenation occurs, resulting in a transient reduction of cellular migration and proliferation, facilitating fibroblast activity in its resident tissues as opposed to following growth factor gradients across the wounded environment. Adaprev may be considered for safe use in the context of flexor tendon surgery, and the use of hypertonic solutions in reducing cell migration and proliferation should warrant further investigation in other tissue types where glide is important for physiological function.

## Supporting Information

Figure S1 **Quantification method for adhesion formation.** A. Quantification of length of adhesion. Arrow denotes adhesion site. A stereological measurement over five equally spaced sections is used to provide an estimation of the adhesion area. B. Quantification of the area of tendon. Arrow denotes adhesion site. A stereological measurement over five equally spaced sections is used to provide an estimation of tendon area. C and D show quantification method for percentage of tendon polarisation. C. Outline of tendon is transferred from standard histology image (B). Note arrow shows adhesion as non polarising area. D. All areas that are polarised are measured to give overall ratio of polarised tendon area to non- polarised tendon area. This is measured over three equally spaced sections and a mean value is calculated. Scale bar represents 200 µm.
(TIF)

Figure S2 **Distribution analysis of DiI injected into wound and sheath space.** Fluorescence microscopy of A. sheath space injected DiI at time point zero. B. sheath space injected DiI at 24 hours. DiI in red. Three dimensional reconstruction of digit showing C. DiI distribution at zero hours and D. DiI distribution at 24 hours. DiI is represented in red, Sheath space is represented in light blue, Bone is represented in orange and subcutaneous tissue is represented in green. Scale bar represents 200 µm.
(TIF)

Figure S3 **Immunohistochemistry of mouse hind paw flexor tendon following surgical laceration.** Cation independent Mannose 6 phosphate receptor (CI-M6PR) expression is shown in A and Transforming growth factor β Receptor 1 (TGFβ-R1) expression is shown in B. Downstream signaling molecules Smad 2 and Smad 3 are shown in C. and D. respectively. Images show longitudinal section of lacerated tendon following. i) shows staining for unwounded controls, all showing evidence of baseline staining in the extracellular matrix and serving as positive controls. After injury PBS treated sham controls (ii) and Adaprev treated tendons (iii) show no evidence of CI-M6PR or TGFβ-R1 expression and no increased expression of downstream Smad 2 or 3. Arrows indicate the point at which the tendon was lacerated and T = tendon. Magnification x10.
(TIF)

Figure S4 **Residency of Adaprev over time in the flexor tendon sheath.** A gradual reduction in bioavailable Adaprev was noted in the flexor sheath with time with 14%, 29%, 28% and 40% decreases in recoverable concentration found at 5, 15, 30 and 45 minutes respectively. Error bars represent standard error of mean. * denotes significant reduction where p<0.05.
(TIF)

Figure S5 **Quantification of “stress shielded” (SS) morphology in cell viability assay.** With 50 mM and 200 mM concentration cells did not show many stress shielded phenotypes until exposure was over 120 minutes (% SS 11.8±1 and 14.1±18 respectively). However 600 mM concentrations number of SS cells increased dramatically to 53.9±2.2, 85.2±8.9 and 97.7±18% with respect to 45 minute, 1 hour or 2 hours worth of exposure. Error bars represent standard error of mean. * denotes significant increase where p<0.05.
(TIF)

Figure S6 **Concentration relationship to Osmolality of Mannose 6-Phosphate.** A linear response to increasing concentration to osmolality was demonstrated.
(TIF)

Figure S7 **Comparison of molecular weight and structure of M6P and G6P.** The molecular weight of the two sugars is identical however G6P has no binding affinity of CI-M6PR.
(TIF)

Figure S8 **Mean migration distance of tendon fibroblasts following treatment with Adaprev and G6P.** Cells did not migrate far without the addition of 10% FBS (45.7 µm versus 278.2 µm). The addition of Adaprev significantly reduced the migration distance of cells (143±29 µm) (p<0.05) whereas G6P also reduced cell migration, this was not found to be significant (202.3±34.5 µm) (p>0.05). Error bars represent standard error of mean.
(TIF)

Figure S9 **Migration of fibroblasts as measured by Cell Titer Glo Luminescence with increasing exposure times to Adaprev or G6P.** Both treatments led to a significant reduction of mean luminescence indicating reduced cell migration with increasing duration of treatment exposure. Error bars represent standard error of mean. Adaprev was significantly more effective at reducing cell migration after 45 and 60 minutes of exposure. * denotes significant difference where p<0.05. Error bars represent standard error of mean.
(TIF)
