# Supplementary figures and images for: Reduction of Tendon Adhesions following Administration of Adaprev, a Hypertonic Solution of Mannose-6-Phosphate: Mechanism of Action Studies
Source: PLoS One. 2014 Nov 10;9(11):e112672. doi: 10.1371/journal.pone.0112672 (PMC4226614; doi:10.1371/journal.pone.0112672)

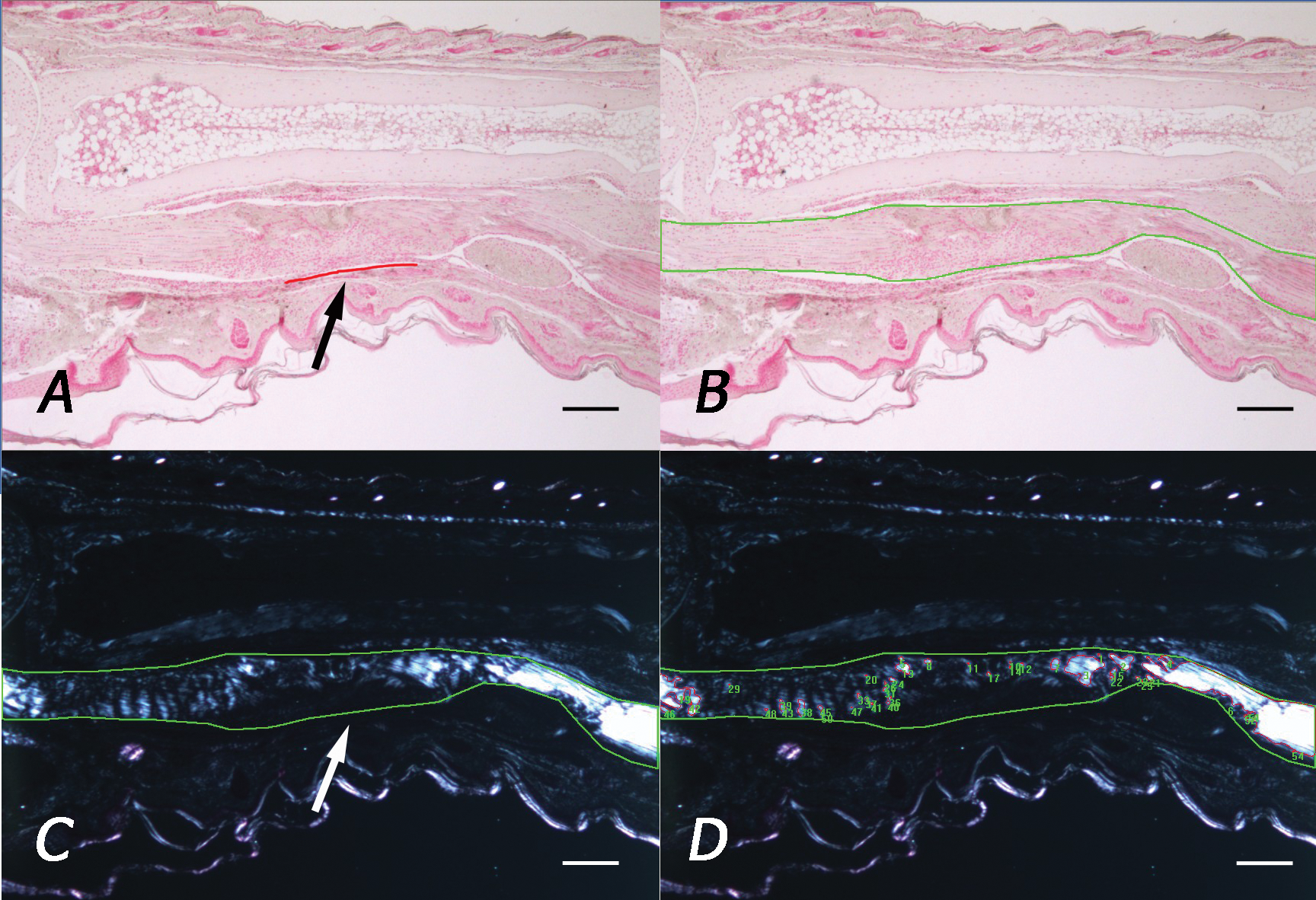

Supplement: Figure S1 — Quantification method for adhesion formation. A. Quantification of length of adhesion. Arrow denotes adhesion site. A stereological measurement over five equally spaced sections is used to provide an estimation of the adhesion area. B. Quantification of the area of tendon. Arrow denotes adhesion site. A stereological measurement over five equally spaced sections is used to provide an estimation of tendon area. C and D show quantification method for percentage of tendon polarisation. C. Outline of tendon is transferred from standard histology image (B). Note arrow shows adhesion as non polarising area. D. All areas that are polarised are measured to give overall ratio of polarised tendon area to non- polarised tendon area. This is measured over three equally spaced sections and a mean value is calculated. Scale bar represents 200 µm. (TIF) [file pone.0112672.s001.tif]

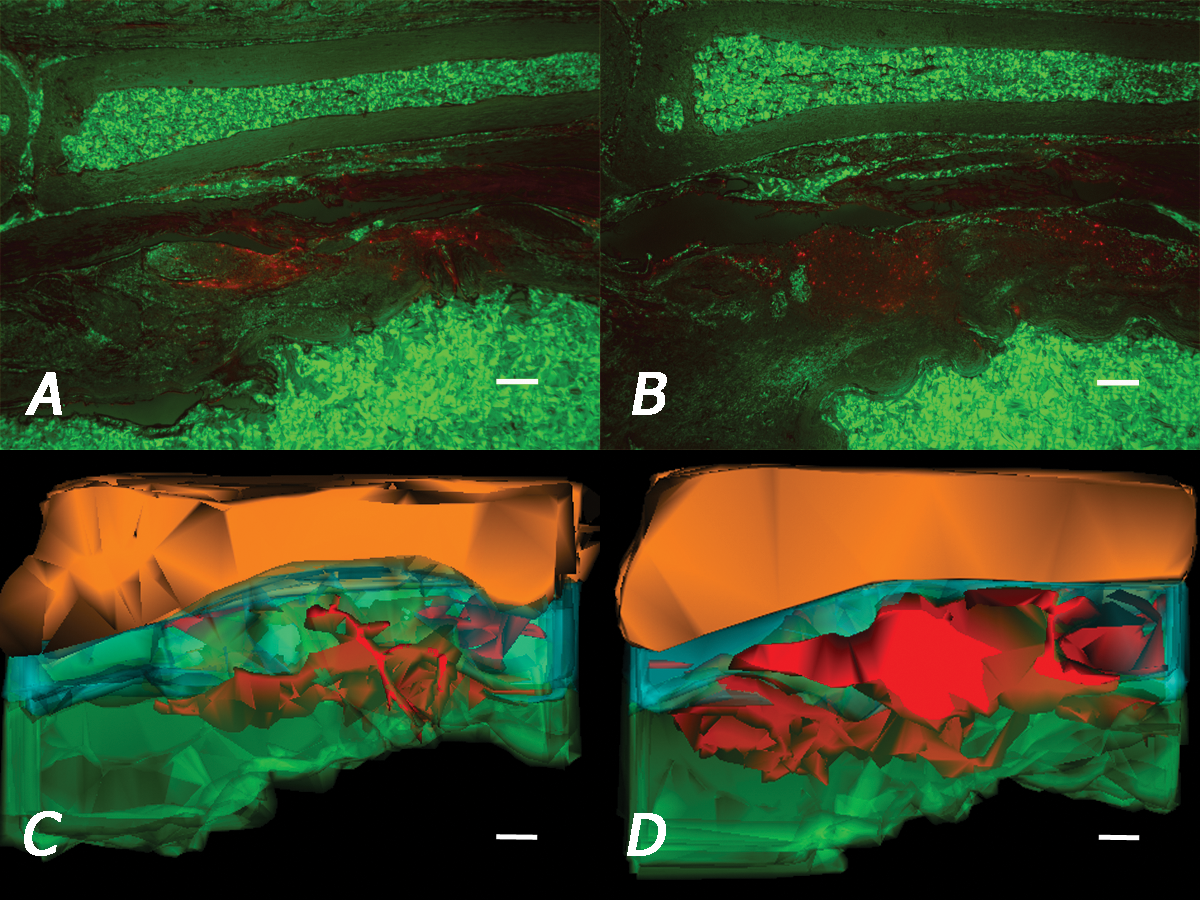

Supplement: Figure S2 — Distribution analysis of DiI injected into wound and sheath space. Fluorescence microscopy of A. sheath space injected DiI at time point zero. B. sheath space injected DiI at 24 hours. DiI in red. Three dimensional reconstruction of digit showing C. DiI distribution at zero hours and D. DiI distribution at 24 hours. DiI is represented in red, Sheath space is represented in light blue, Bone is represented in orange and subcutaneous tissue is represented in green. Scale bar represents 200 µm. (TIF) [file pone.0112672.s002.tif]

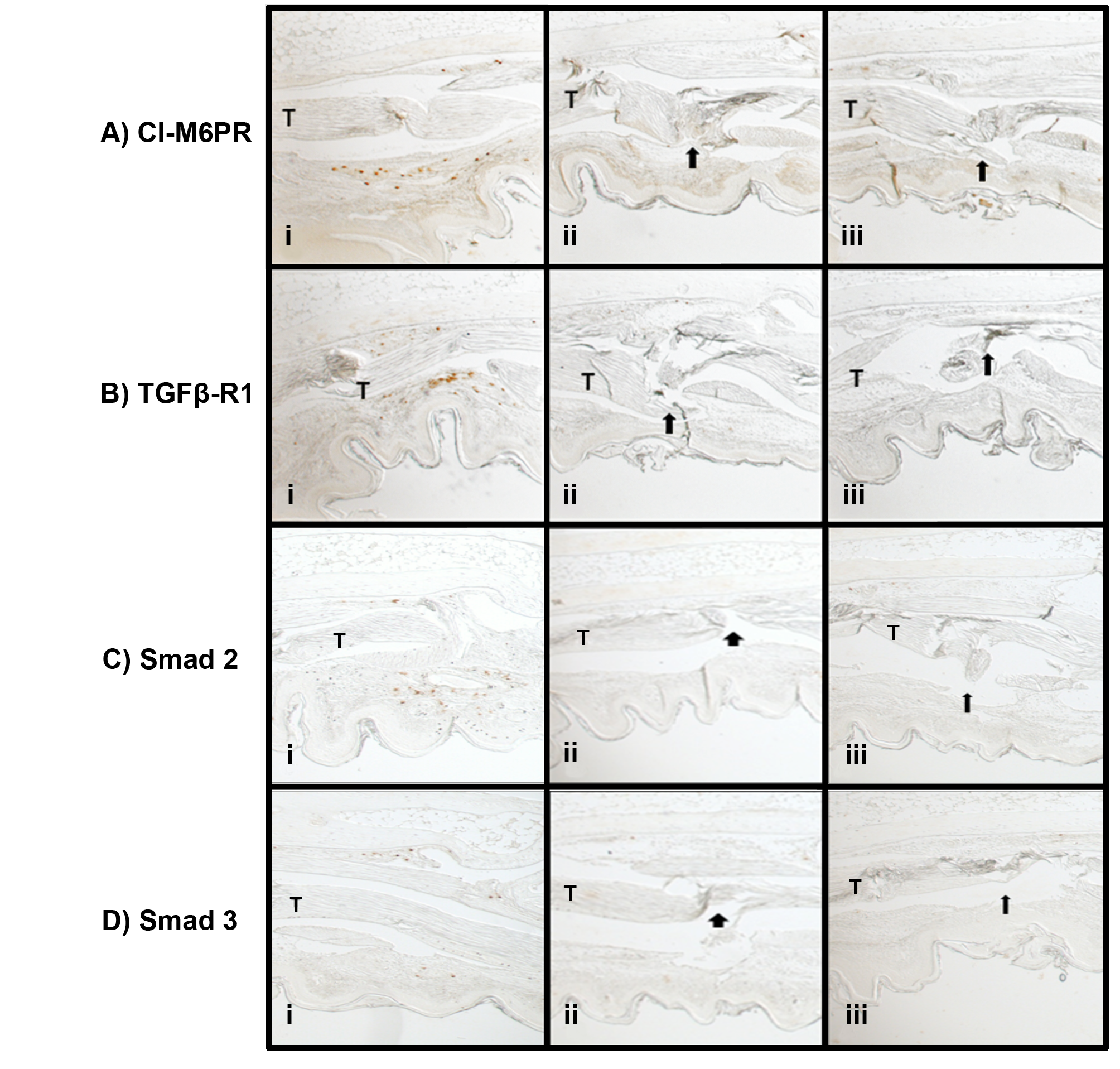

Supplement: Figure S3 — Immunohistochemistry of mouse hind paw flexor tendon following surgical laceration. Cation independent Mannose 6 phosphate receptor (CI-M6PR) expression is shown in A and Transforming growth factor β Receptor 1 (TGFβ-R1) expression is shown in B. Downstream signaling molecules Smad 2 and Smad 3 are shown in C. and D. respectively. Images show longitudinal section of lacerated tendon following. i) shows staining for unwounded controls, all showing evidence of baseline staining in the extracellular matrix and serving as positive controls. After injury PBS treated sham controls (ii) and Adaprev treated tendons (iii) show no evidence of CI-M6PR or TGFβ-R1 expression and no increased expression of downstream Smad 2 or 3. Arrows indicate the point at which the tendon was lacerated and T = tendon. Magnification x10. (TIF) [file pone.0112672.s003.tif]

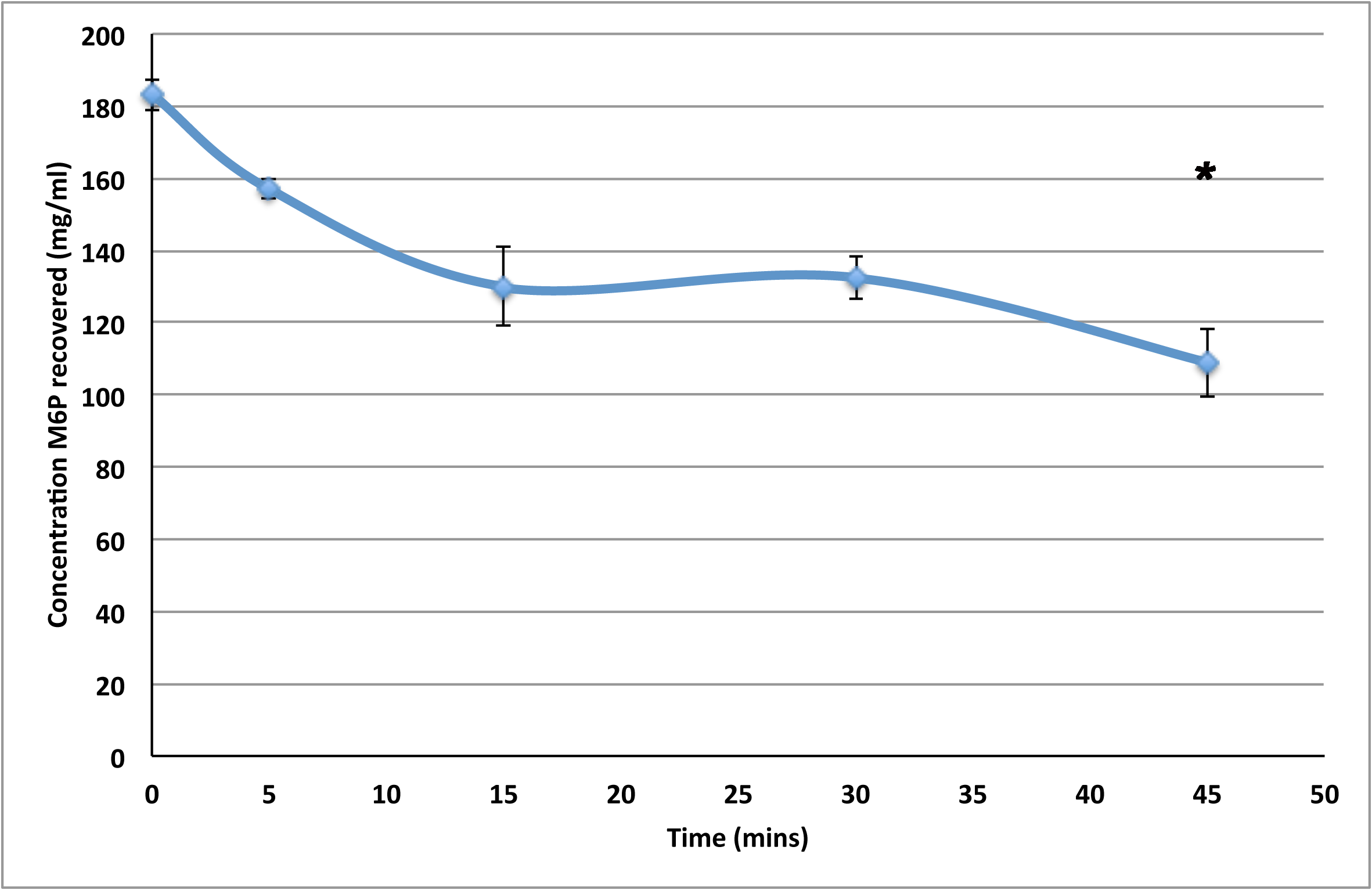

Supplement: Figure S4 — Residency of Adaprev over time in the flexor tendon sheath. A gradual reduction in bioavailable Adaprev was noted in the flexor sheath with time with 14%, 29%, 28% and 40% decreases in recoverable concentration found at 5, 15, 30 and 45 minutes respectively. Error bars represent standard error of mean. * denotes significant reduction where p<0.05. (TIF) [file pone.0112672.s004.tif]

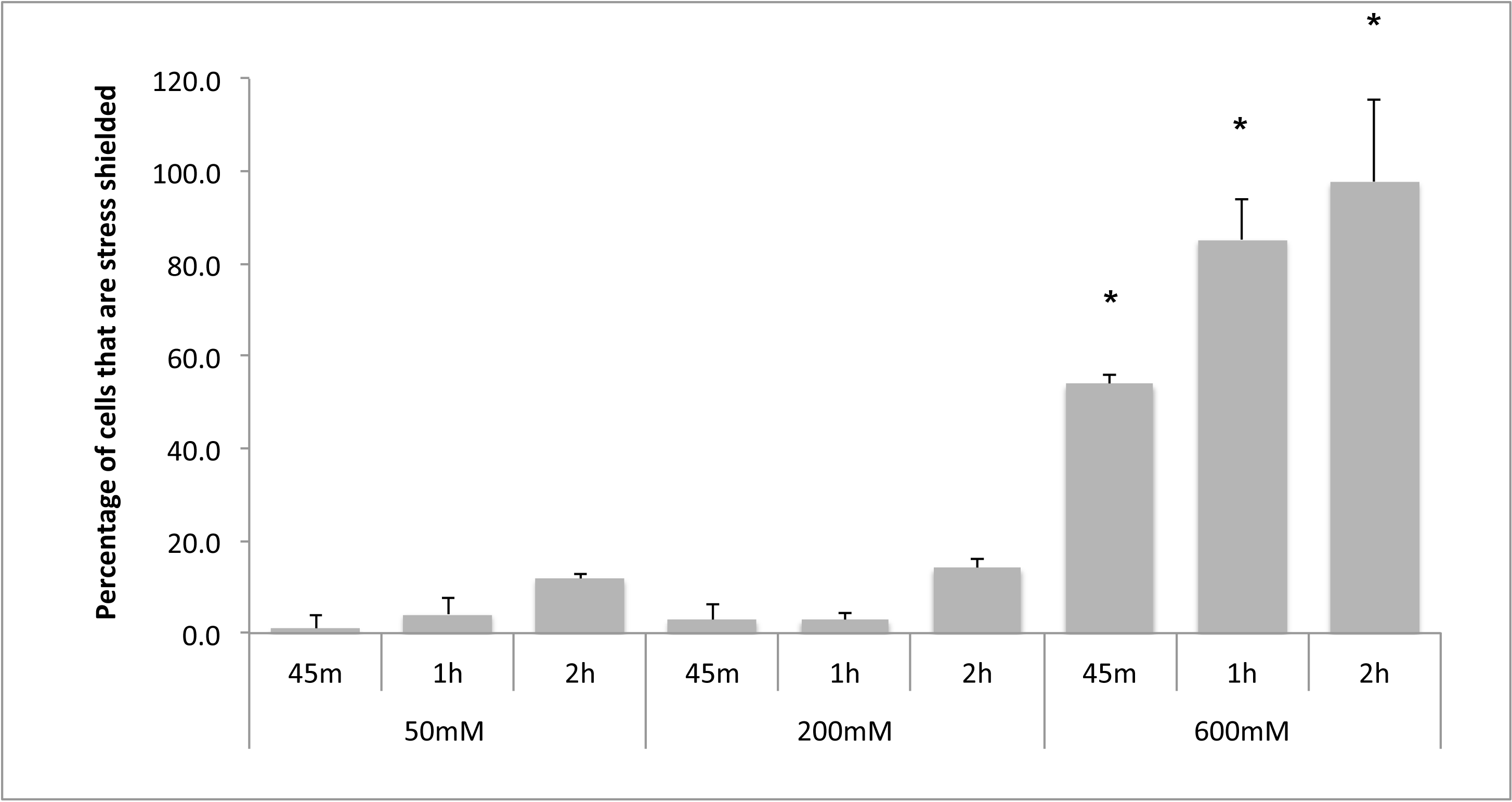

Supplement: Figure S5 — Quantification of “stress shielded” (SS) morphology in cell viability assay. With 50 mM and 200 mM concentration cells did not show many stress shielded phenotypes until exposure was over 120 minutes (% SS 11.8±1 and 14.1±18 respectively). However 600 mM concentrations number of SS cells increased dramatically to 53.9±2.2, 85.2±8.9 and 97.7±18% with respect to 45 minute, 1 hour or 2 hours worth of exposure. Error bars represent standard error of mean. * denotes significant increase where p<0.05. (TIF) [file pone.0112672.s005.tif]

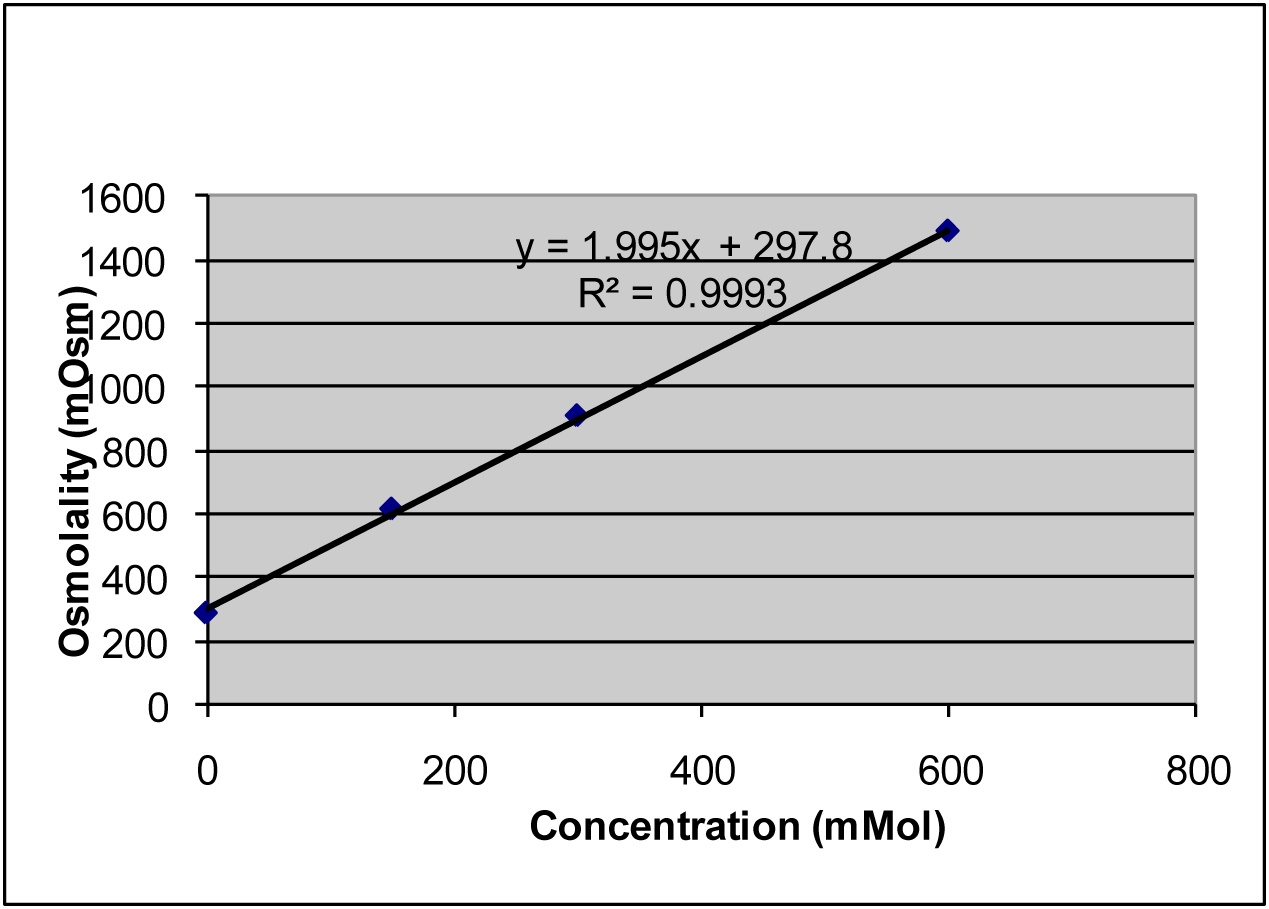

Supplement: Figure S6 — Concentration relationship to Osmolality of Mannose 6-Phosphate. A linear response to increasing concentration to osmolality was demonstrated. (TIF) [file pone.0112672.s006.tif]

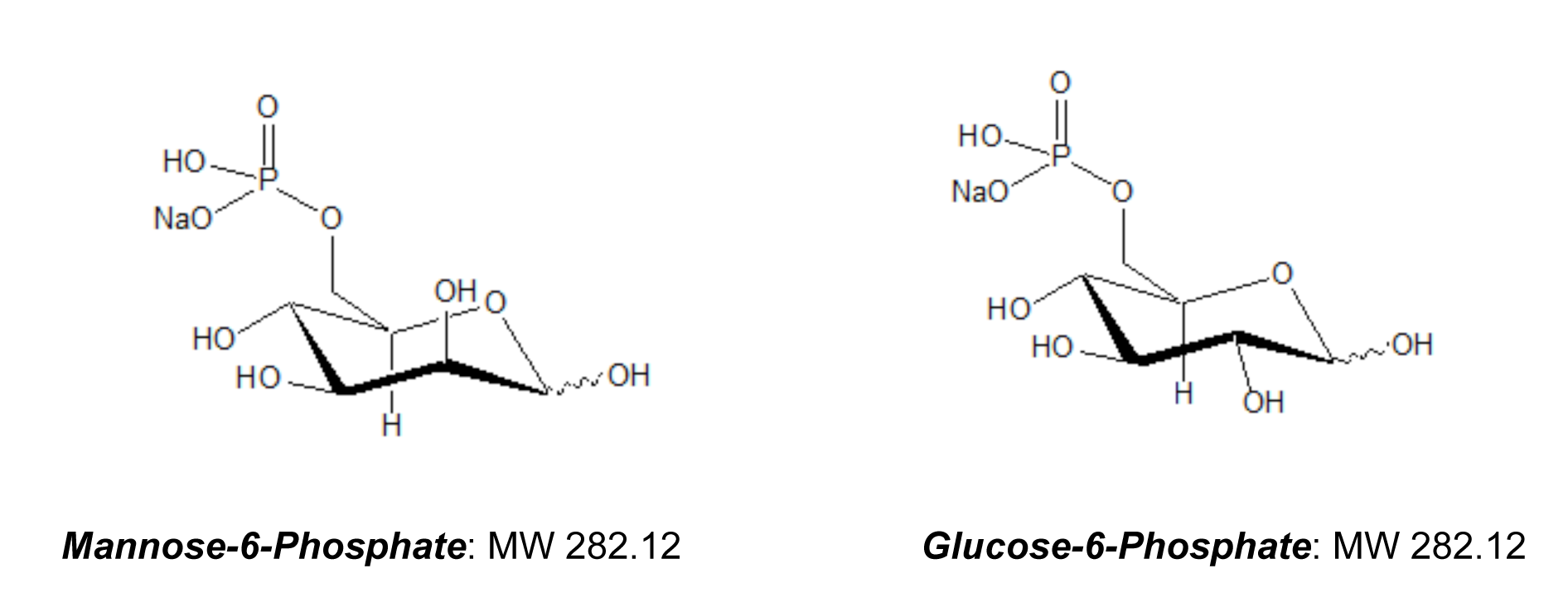

Supplement: Figure S7 — Comparison of molecular weight and structure of M6P and G6P. The molecular weight of the two sugars is identical however G6P has no binding affinity of CI-M6PR. (TIF) [file pone.0112672.s007.tif]

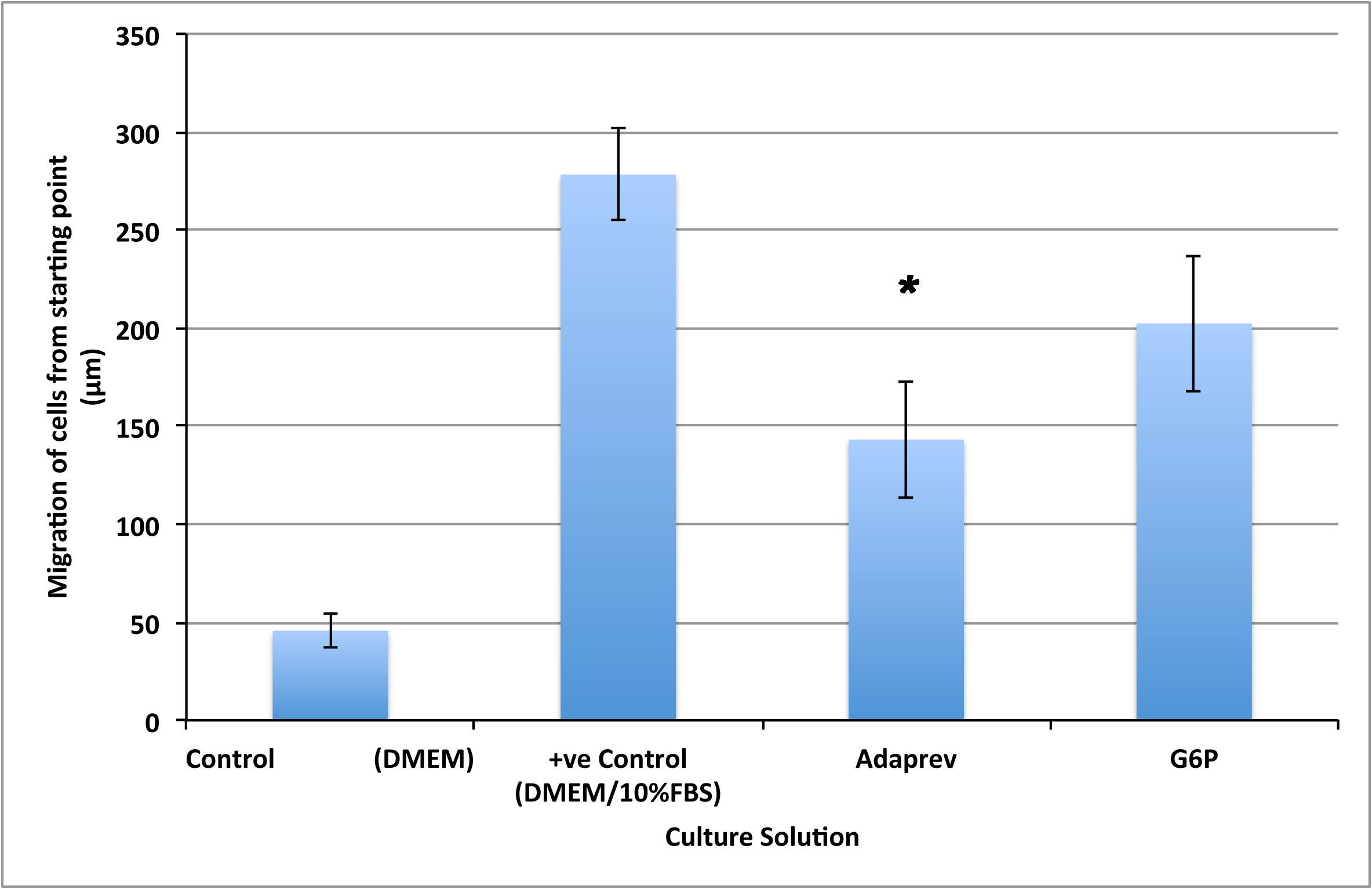

Supplement: Figure S8 — Mean migration distance of tendon fibroblasts following treatment with Adaprev and G6P. Cells did not migrate far without the addition of 10% FBS (45.7 µm versus 278.2 µm). The addition of Adaprev significantly reduced the migration distance of cells (143±29 µm) (p<0.05) whereas G6P also reduced cell migration, this was not found to be significant (202.3±34.5 µm) (p>0.05). Error bars represent standard error of mean. (TIF) [file pone.0112672.s008.tif]

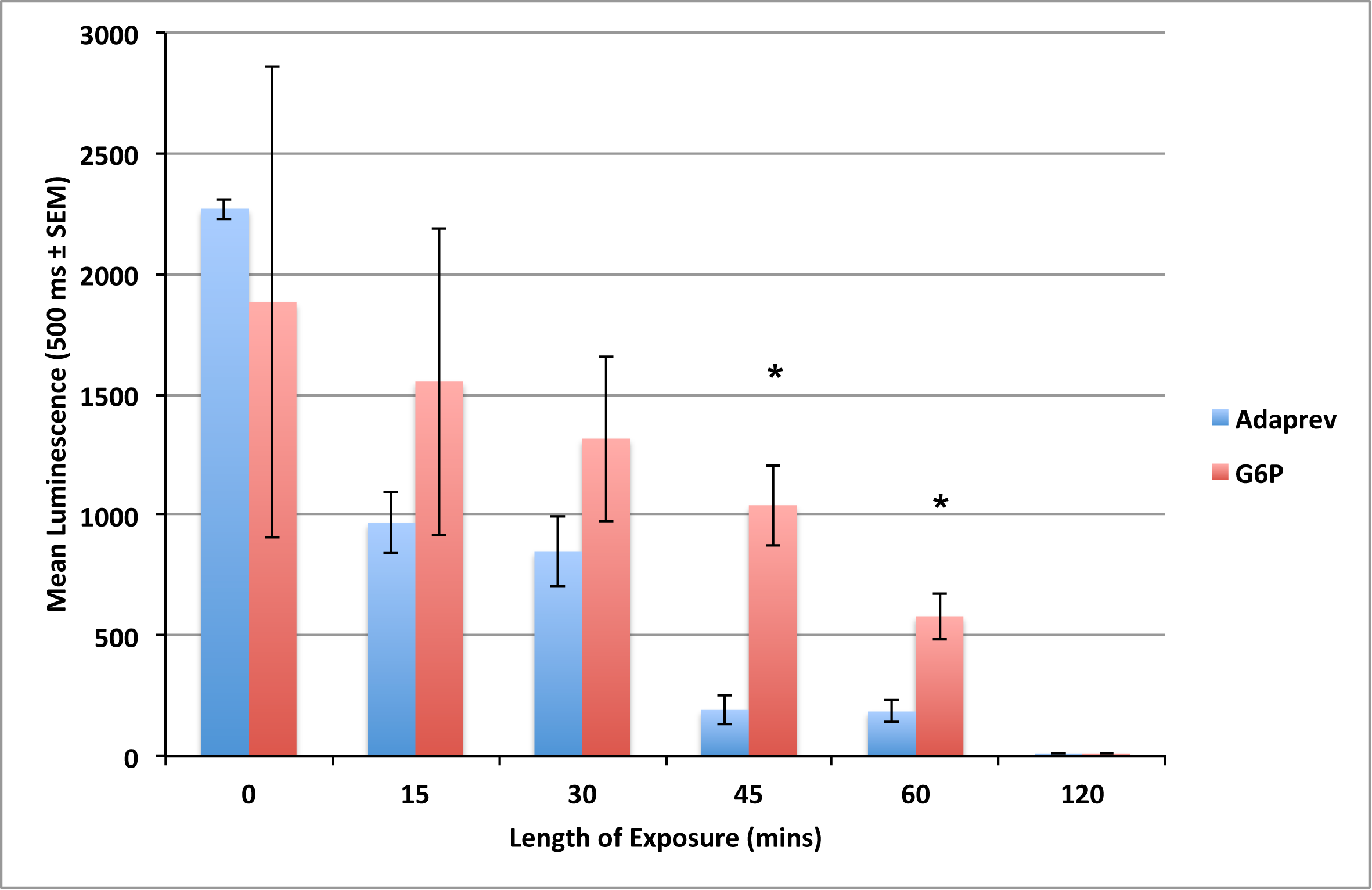

Supplement: Figure S9 — Migration of fibroblasts as measured by Cell Titer Glo Luminescence with increasing exposure times to Adaprev or G6P. Both treatments led to a significant reduction of mean luminescence indicating reduced cell migration with increasing duration of treatment exposure. Error bars represent standard error of mean. Adaprev was significantly more effective at reducing cell migration after 45 and 60 minutes of exposure. * denotes significant difference where p<0.05. Error bars represent standard error of mean. (TIF) [file pone.0112672.s009.tif]
